# Goosefoot Oil in the Treatment of Round Worms

**Published:** 1909-01-16

**Authors:** 


### GOOSEFOOT OIL IN THE TREATMENT OF ROUND WORMS.

The ethereal oil prepared from *Chenopodium anthelminticum*, a plant which grows wild in the United States (oleum chenopodii anthelmintici æthereum) has been long known as a remedy for worms, but has been little used in Europe. It has been shown by Brüning and others that although it will not expel oxyuris vermicularis, trichocephalus dispar, or ankylostomum duodenale, it is almost a specific for ascarides or round worms; it is not difficult to take, and it is said to act promptly and without unpleasant secondary actions. The oil is a yellow fluid of a powerful aromatic odour; left exposed to air, the colour darkens in time. It may be administered in drops with sugar and water, or an emulsion may be prescribed.

